# Biological Monitoring of Glyphosate Exposure among Knapsack Sprayers in Khon Kaen, Thailand

**DOI:** 10.3390/toxics12050337

**Published:** 2024-05-06

**Authors:** Sunisa Chaiklieng, Kodchakorn Uengchuen, Netsirin Gissawong, Supalax Srijaranai, Herman Autrup

**Affiliations:** 1 Department of Occupational Safety and Environmental Health, Faculty of Public Health, Khon Kaen University, Khon Kaen 40002, Thailand; 2 Program in M.Sc. Occupational Health and Safety, Faculty of Public Health, Khon Kaen University, Khon Kaen 40002, Thailand; 3 Materials Chemistry Research Center, Department of Chemistry and Center of Excellence for Innovation in Chemistry, Faculty of Science, Khon Kaen University, Khon Kaen 40002, Thailand; 4 Institute of Public Health, Aarhus University, 8000 Aarhus, Denmark

**Keywords:** biomatrix, urinary glyphosate, risk assessment, occupational exposure

## Abstract

Sprayers’ exposure to glyphosate was analyzed through detection of its biomarker in spot urine biological monitoring, and the health risk was assessed using the biomatrix model. Urine samples were collected from 15 sprayers after spraying, and the glyphosate concentration was determined by using the DLLME-HPLC method with a UV detector. The calibration curve for glyphosate was linear in the range of 0.4–100 µg/L, while the limits of detection and quantification were 0.1 µg/L and 0.4 µg/L, respectively. The human health risk was estimated using the hazard quotient (HQ) and the biomatrix of risk assessment. The internal dose ranged from 0.0001 to 0.0021 mg/kg b.w./day. The non-cancer HQ showed no potential health risk concerns (HQ < 1). The biomatrix of health risk assessment, based on urinary glyphosate concentration, exhibited a strong correlation with the health risk matrix model. This correlation was determined by considering the likelihood of exposure, calculated from the quantity of glyphosate used and the usage of personal protective equipment (r = 0.854, *p* < 0.001). Although low risk was observed in sprayers, proper PPE use and the application of more knowledge are required. The simplified health risk assessment can be used for easy self-assessment of risk in preventive action regarding health risk awareness among sprayers.

## 1. Introduction

Glyphosate, N-(phosphonomethyl) glycine, is a nonselective herbicide that has been extensively used worldwide for weed control in residential and agricultural areas [1,2]. In 2015, the International Agency for Research on Cancer (IARC) classified glyphosate as a Group 2A substance, probably carcinogenic to humans [3]. Glyphosate alone was found to have the lowest toxicity in animal studies [4]; however, the first generation of polyethoxylated amine (POEA) surfactants in Roundup were found to be markedly more toxic than glyphosate, and this heightened concerns about risks to human health, especially among applicators [5].

Thailand’s agricultural system and land uses, like those of most Southeast Asian countries, have shifted from traditional crop cultivation to commercial crop cultivation. In terms of pesticide use per unit area, Thailand ranks third out of 15 Asian countries [6]. Pesticide use has increased in Thailand as agricultural systems have changed. It is possible that farmers’ lack of education contributes to pesticide overuse. Most farmers have only completed primary school, making it difficult for them to comprehend technical terms and complicated label instructions [7,8,9]. Farmers typically choose pesticides based on recommendations from neighbors, television commercials, and retailers [7,8,9]. According to the cases from occupational disease surveillance systems reported by hospitals, pesticide poisoning among the farmers of northeastern Thailand resulted in a morbidity rate of 35.6 per 100,000 people in 2016, rising from 25.3 per 100,000 people in previous years [10]. Because some symptoms associated with pesticide exposure are nonspecific (i.e., headache, dizziness, nausea, vomiting, cramps, muscular weakness, etc.), primary healthcare providers may be unable to diagnose pesticide poisoning. Thus, the less severe cases were not included in the records [7,10].

For non-cancer toxicant risk assessment, the hazard quotient (HQ) is used to measure the ratio of potential exposure to a chemical and the level at which no adverse effects are expected, and it is found by dividing the exposure dose by a reference dose (RfD). The U.S. EPA established the Reference Dose (RfD) Work Group in order to clarify the use of experimental evidence to show that a substance is not carcinogenic or mutagenic when making regulatory choices about the significance of chemical exposures; the RfD of glyphosate, according to the standard report on health risk assessment of glyphosate, is 1 mg/kg b.w./day [11]. An HQ of less than 1 is considered acceptable, while an HQ value of greater than 1 suggests a possible risk to human health [12]. There are various methods for determining glyphosate exposure concentrations [13]. To enhance the sensitivity of the detection of chemicals, preconcentration methods have been developed for the determination of substances at trace levels. Dispersive liquid–liquid microextraction (DLLME) is a popular sample preparation technique and has been widely used to extract various compounds [14].

The measurement of urinary glyphosate in farmers is considered a marker of exposure. Therefore, the purpose of this study was to investigate exposure to glyphosate among sprayers through biological monitoring for health risk assessment. The objective was to obtain values for risk-group screening and estimate the potential health risks associated with occupational exposure to glyphosate.

## 2. Materials and Methods

### 2.1. Participants

A total of 15 male farmers were recruited from the previous study conducted during the primary phase of the research, which aimed to assess the occupational health risk of glyphosate exposure among sprayers from 12 sub-districts of Nampong district, Khon Kaen province [15]. The recruitment took place between November and December 2020. The district is predominantly an agricultural area, with the production of rice and sugar cane, as well as livestock farming, as its primary industries. Only participants whose glyphosate spraying activities were >1 h/day spraying and at least 100 mL/year application (48% *w*/*v* glyphosate concentration in Roundup) were included in the study. Farmers provided written informed consent. The project was approved by the Khon Kaen University Ethics Committee in Human Research (HE 612297).

The participants completed a project questionnaire and provided one urine sample (15 mL) 4 h after spraying [16]. A questionnaire was used to collect information on participant demographics, health effects, application information, and use of personal protective equipment [15]. All urine samples were frozen at −20 °C within 24 h of collection and kept at this temperature until laboratory analysis.

### 2.2. Determination of Glyphosate in Urine Samples

**Figure 1:**
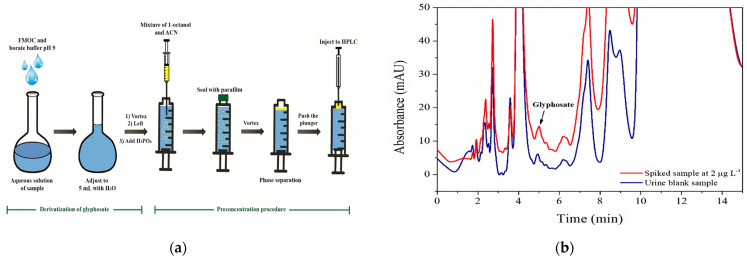
(**a**) Schematic diagram illustrating in-syringe-based DLLME for the extraction of glyphosate; (**b**) chromatograms of urine samples; blank sample (blue) and spiked sample (red) with 2 µg/L of glyphosate.

All urine samples were deproteinized and cleaned up prior to DLLME-HPLC analysis. Each urine sample (500 uL) was deproteinized and cleaned up using 500 μL of acetonitrile (ACN) and 1.14 mg of a strong anion-exchanger resin (amberlite IRA-900 in chloride form). Glyphosate was eluted from the resin with 2 mL of a mixture of ACN and 1 mol/L NaCl (50:50 *v*/*v*). The eluate was then analyzed by DLLME-HPLC. To facilitate detection by UV absorption after the HPLC analysis, glyphosate underwent pre-column derivatization using 9-fluorenyl methoxycarbonylchloride (FMOC). In addition, DLLME was employed to enrich the glyphosate, so trace amounts of glyphosate could be reliably detected. The derivatization of glyphosate combined with the in-syringe DLLME method previously described by Laosuwan et al. [17] is illustrated in Figure 1a. The deproteinized sample solution (2 mL) was derivatized with FMOC (5 g/L, 50 μL) in borate buffer, pH 9. After 30 min, 150 µL of 10% (*v*/*v*) H_3_PO_4_ was added, and the resulting glyphosate-FMOC solution was transferred to a 10 mL plastic syringe. The solution containing the extraction solvent (75 µL of 1-octanol) and the dispersive solvent (200 µL of ACN) was rapidly injected into the syringe containing glyphosate-FMOC. The extraction phase (the upper phase) was diluted with 5 µL of ACN before it was analyzed by HPLC using an Inertsil C4 (5.0 µm, 150 mm × 4.6 mm i.d) column (Inertsil, GL Sciences Inc., Tokyo, Japan) with mobile phases of ACN and 1% H_3_PO_4_ (30:70 *v*/*v*) under isocratic elution at a flow rate of 1.0 mL min^–1^ and detection at 206 nm. The linear range was in the range of 0.4–100 µg/L with intraday and interday coefficients of variation (CVs) of 4.5% (*n* = 5) and 8.8% (*n* = 15, over 3 runs), respectively. The deduced limit of detection (LOD) and quantification (LOQ), based on signal-to-noise ratios (S/N) of 1:3 and 1:5, were 0.1 µg/L and 0.4 µg/L, respectively. The typical chromatograms of a urine sample (blank) and a urine sample spiked with glyphosate standard are displayed in Figure 1b, and it can be seen that the glyphosate peak (at a retention time of 5.1 min) is clearly observed among the peaks of matrices. In addition, the result was confirmed by the spiked sample peak (Figure 1b).

### 2.3. Urinary Glyphosate Concentration and Internal Dose Calculation

The amount of urinary glyphosate, which is considered to be an indicator of the internal dose, can be calculated using Equation (1) below:

Internal (systemic) dose = C_urine_ × V_urine_/b.w., (1)

where C_urine_ = urinary glyphosate concentration (µg/L), V_urine_ = daily urinary output (L per day), and b.w. = body weight (kg). The urine output determined is 2000 mL per day, depending on the body weight (b.w.; in kg) of a given individual [18,19].

### 2.4. Risk Assessment

#### Hazard Quotient (HQ) Calculation

A hazard quotient (HQ) is the ratio of the potential exposure to a selected C_urine_, relative to the level at which no adverse effects are expected. After the internal dose is computed, HQ can be obtained using Equation (2) [12]:

HQ = Internal dose/RfD,(2)

### 2.5. Health Risk Assessment (HRA) Matrix and HRA Biomatrix (HRAB) Models

The health risk assessments from the previous study [16] and the health risk assessment using the biomatrix model [20] are compared below. The models differ in terms of exposure assessment: in the former, four levels are estimated based on the amount of glyphosate exposure per activity multiplied by frequency per year, and PPE used [18], while the latter consists of four levels of scoring classification for the concentration of glyphosate in urine (C_urine_) (μg/L), as shown in Table 1. In this study, the HRAB applied by Chaiklieng et al. [20], a risk matrix (5 × 4) of the likelihood of exposure levels, the concentration of C_urine_ (μg/L), and the severity levels of adverse symptoms were used to perform the steps of the HRAB model, as shown in Table 1. There are three steps in the HRAB model of health risk assessment. The first step is the classification of the exposure level according to C_urine_ concentration (μg/L). The score or the level for the concentration of C_urine_ is determined according to the quartile classification level [21]: level (score of) 1: <0.1 µg/L, level 2: 0.1–5.83 μg/L, level 3: 5.83–12.11 μg/L, and level 4: >12.11 μg/L. The second step is the classification of the severity of adverse effects experienced during the previous 6 months of glyphosate application, which was divided into 4 levels: (1) no symptoms; (2) mild symptoms; (3) moderate symptoms; and (4) severe symptoms. Multiplying the likelihood levels by the severity levels of adverse symptoms, which determines the risk scores for determining overall risk levels, is the third step in the HRAB model. In order to get the final risk level estimation, which was broken down into four categories as the final step (Table 1), the HRAB approach established numerical ranges for various criteria of individual scores. These four categories were: score of 1–3 (acceptable health risk), score of 4–8 (low health risk), score of 9–12 (moderate health risk), and score of 13–16 (high health risk).

In the previous study’s HRA matrix model [15], a risk matrix (5 × 4) of the likelihood of exposure levels via scoring of the glyphosate volume used per year and PPE usage, and the adverse symptoms level were used to perform the health risk assessment. There were four steps of risk assessment in the risk matrix model for evaluating health risk. Firstly, the four levels (scores) of the pure glyphosate volume used by the sprayers were classified as follows: (1) up to 100 mL; (2) >100–499 mL; (3) 500–1000 mL; and (4) >1000 mL. Secondly, the score for PPE usage, which was divided into four categories (0, 1, 2, 3) based on the descriptions shown in Table 1, was added to the score level of glyphosate (1, 2, 3, 4). We determined the likelihood of exposure by considering the PPE usage and the glyphosate use scores. Subsequently, in the third step, the severity levels of adverse symptoms were classified as mentioned above and used in another approach of the risk assessment matrix model by multiplying them with the likelihood levels for health risk estimation.

The correlation between the overall risk data of the HRAB and the HRA matrix models was compared using the difference in likelihood approach. Long-term health effects are generally assessed using a HRA biomatrix (HRAB) model for health risk assessment.

### 2.6. Data Analysis

Urine samples were classified by using descriptive analysis for general information and were ranked from lowest to highest, and the thresholds for each quartile, Q1–Q2, Q2–Q3, and Q3–Q4, were defined by the values corresponding to the 25th, 50th, and 75th percentiles of the distribution, respectively. In addition, percentage mean and standard deviation (SD), as well as maximum and minimum values, were used. The Spearman’s rank correlation coefficient was used to indicate the correlation between internal (systemic) dose levels and the health risk assessment matrix levels. This analysis was performed using STATA (Brazos County, TX, USA).

## 3. Results

### 3.1. Descriptive and Summary Statistics of Demographic and Task Characteristics

Table 2 details the selected characteristics of participants based on information reported in their enrollment and follow-up questionnaires. All participants were male, and their mean (SD) age was 53.40 (8.39) years. Their maximum and minimum body weights were 70 and 49 kg, respectively, while the average weight (SD) was 58.67 (5.17) kg. In total, 70 percent of individuals had completed either primary school or junior high school, while 26.7% had completed senior high school. Most participants had an average spraying experience of 16.47 (SD = 7.52) years. The mean (SD) frequency of spraying and the duration of spraying per activity per year were 17.07 (SD = 16.90) times and 180 (143.43) min, respectively. The amount of glyphosate mixed and sprayed per year ranged from 100 to 1000 mL. Approximately 86 percent of individuals sprayed in the direction of the lower knees and in a non-directional position (13.3%). Wind direction was observed every time by 80% of participants, occasionally by 6.6%, and never by 13.3%. Among the participants, 12 reported clear weather and three reported cloudy weather while spraying.

### 3.2. Relationship between Behavior in Using Personal Protective Equipment (PPE) and Symptoms Associated with Pesticide Spraying

The relationship between personal protective equipment (PPE) use and symptoms was analyzed based on the questionnaire information regarding the previous 6 months of spraying. The types of individual protective equipment used by participants in the symptoms group were rubber gloves, cotton masks, boots, long-sleeved shirts, and long pants. Glasses and hats were seldom used, as shown in Table 3.

### 3.3. Personal Hygiene of Participants with Symptoms Associated with Pesticide Spraying

Three participants who ate in the spraying area experienced symptoms during the previous 6 months of spraying. Although some had good personal hygiene, such as washing hands and showering 1 h after work, the percentages of participants who performed such actions and expressed symptoms were 100% and 40%, respectively. After spraying, five participants with contaminated clothes showed symptoms. Among the 14 participants who had cleaned backpack sprayers, after spraying, nine participants did not report symptoms, while six participants had mild symptoms (headache and dizziness). Only one participant had moderate symptoms (nausea), as shown in Table 4.

### 3.4. Glyphosate Concentration in Urine Samples (C_urine_)

In the analysis of glyphosate in urine samples (*n* = 15), nine samples (60%) had a detectable glyphosate concentration: median = 19.42 μg/L. The maximum glyphosate level found in urine was 57.49 μg/L. Among the remaining samples, the glyphosate concentrations were 45.42, 20.85, 12.63, 11.58, 11.39, 5.9, 5.83, and 3.66 μg/L, respectively. Six participants had a detected C_urine_ level lower than the LOD, which was 0.1 µg/L.

This study divided C_urine_ into four quartiles of urine concentration, which were as follows: less than or equal to 0.1 µg/L (level 1), above 0.1 to 5.83 μg/L (level 2), above 5.83 to 12.11 μg/L (level 3), and above 12.11 μg/L (level 4), as described in Table 1.

### 3.5. Internal (Systemic) Doses and Hazard Quotient

The glyphosate exposure was calculated from the internal dose. The C_urine_ values were used in calculations for internal dose estimations. The average internal dose of sprayers, via all exposure routes while working, was calculated after spraying for 4 h and found to be 0.00004 mg/kg b.w./day, with a range of 0.00001 to 0.00021 mg/kg b.w./day.

The relevant RfD was determined by using the U.S. EPA standard for glyphosate and was based on an RfD of 1 mg/kg b.w./day [11]. According to the HQ values of sprayers, 100% of them had an acceptable level of non-carcinogenic risk (HQ < 1).

### 3.6. Biomatrix Risk Assessment Model of Glyphosate

The vast majority of participants had a likelihood of exposure at level 2 (86%) or level 3 (13%) according to the yearly amount of glyphosate applied per year and PPE usage (Table 1) estimated by the health risk assessment model. As demonstrated in Table 5, the largest proportion of participants (six persons) had a low (level 1) likelihood of exposure according to the biomatrix risk assessment model, followed by the proportions at level 4 (26%), level 3 (20%), and level 2 (13%). The result of likelihood of exposure (quantity of glyphosate used and PPE usage) and severity of symptoms experienced during the previous 6 months of spraying showed that the risk was acceptable (*n* = 9) or low (*n* = 6), according to the health risk assessment model. Similarly, the largest proportion of participants had an acceptable risk (*n* = 9), followed by the proportion at low risk (*n* = 6), based on the exposure according to C_urine_ in the biomatrix model (Table 5). A comparison between risk assessments using the quantity of glyphosate used and PPE usage, and C_urine_ showed that risk levels were statistically correlated according to the Spearman’s rank correlation coefficient of r = 0.85 at *p* < 0.001.

## 4. Discussion

In this study of 15 participants, nine sprayers had detectable levels of glyphosate in urine, while six had levels lower than the LOD. However, analysis of sprayers with no detected glyphosate revealed that the frequency of spraying was 60 times per year, with only a small volume of glyphosate used. The urinary glyphosate concentrations in the present analysis (3.66–57.49 µg/L) resembled those of other reports on glyphosate exposure among sprayers [22]. It was reported that baseline urinary glyphosate concentrations among forest workers spraying Roundup (8% Roundup-containing solution) in Finland were <100 µg/L [23]. In Ireland, amenity horticulturists were analyzed before and after spraying, and a pre-spraying mean of 1.20 µg/L, a post-spraying mean of 1.72 (1.53) µg/L, and a peak sample of 2.53 (1.89) µg/L were found [24]. These results were consistent with the study of Khangkhun et al. (2020) [23] in Thailand, which found that the sprayers had a lower geometric mean (GM) of urinary glyphosate concentration before application (22.4 µg/L) compared with after application (47.1 µg/L) and the morning following the application (49.49 (2.63) μg/g creatinine) [21]. The current study revealed a higher C_urine_ among participants (57.49 µg/L) compared with farmers in northern Thailand (13.30 µg/L) [24].

Urinary glyphosate concentrations were highest in samples taken up to 3 h after work [18] and were in agreement with the biological half-life of glyphosate, which is approximately between 3 ½ and 14 ½ h [24]. The concentrations of glyphosate in the previous study were found to be in quartile 4 of the current study. Application of glyphosate-containing products produces only a transient effect on urinary glyphosate levels as glyphosate is rapidly excreted from the body [25].

All of the participants wore a protective mask, a long-sleeved shirt, and long pants. To prevent skin exposure, the majority of participants wore gloves and boots during spraying. Long-sleeved shirts were important in the prevention of glyphosate exposure during application among sprayers [26]. The study by Taneepanichskul et al. [27] found that farmers used rubber gloves for farming, but approximately 80 percent of the farmers had never washed or cleaned their gloves and reused them after using pesticides. The study of Wongwichit et al. [28] revealed that maize farmers in Nan province of northern Thailand did not use PPE during herbicide application. Similarly, in South Africa, workers were found to have low PPE compliance. The data revealed that workers either did not have access to a filter mask or did not want to wear one in the hot weather conditions, indicating use was unbearable [29]. Further research is required to determine how to establish a robust correlation between PPE and adverse effects in a large population.

In Phayao province, northern Thailand, where PPE was always used during application of pesticides, it was found that the most used PPE types were long-sleeved shirts and long pants and boots, while masks, gloves, and goggles were used the least [7,30]. However, cheap PPE that did not comply with the chemical protection standard was commonly provided to sprayers. Thailand’s farmers may face financial difficulties in purchasing standard PPE. Some of the PPE used for pesticide exposure protection was inappropriate. Some long-sleeved shirts, long pants, and masks were made of a fabric that allows pesticides to pass through the skin and into the lungs through inhalation [31].

All sprayers in the study wore masks, although various types were used. The sprayers with a cotton mask showed the highest exposure levels. Improper use of PPE may increase the risk of glyphosate penetration into the skin.

In this study, nine of the participants reported symptoms associated with pesticide poisoning, which were dizziness, headache, skin irritation, rash, numbness, and dry lips. A study of rice farmers in northern Thailand found that those who scattered seed and harvested the crops had a higher prevalence of numbness in their hands or feet [7]. The symptoms reported in vegetable farmers from northeastern Thailand were neurologic (80 percent), neuromuscular (75 percent), optical (50 percent), and gastrointestinal (45 percent), whereas the symptoms in rice farmers from central Thailand were neuromuscular (62.9 percent), respiratory (41.9 percent), dermatologic (21.9 percent), gastrointestinal (20.7 percent), and optical (18.6 percent) [32,33]. In India, it was found that long-term exposure to pesticides among professional pesticide sprayers resulted in a higher prevalence of breathlessness (odds ratio [OR]: 3.14, 95% confidence interval [CI]: 1.22–8.07), chronic cough/phlegm (OR: 3.53, 95% CI: 1.09–11.46), symptoms of peripheral sensory neuropathy (OR: 6.66, 95% CI: 2.53–17.51), and recurrent abdominal pain (OR: 3.05, 95% CI: 1.03–9.01) when compared to people engaged in other occupations [32,34]. In Sri Lanka, it was reported for the first time that 39 new vulnerable CKDu cases had been discovered in sugarcane farming areas. Renal function markers in sugarcane farmers were lower, while urinary biomarkers of tubular injury, KIM-1 and NGAL, were higher, both of which indicate diminishing kidney function. Glyphosate was significantly correlated with ACR, eGFR, and NGAL [35].

Sprayers used more than just glyphosate to complete the task; therefore, symptoms associated with glyphosate poisoning were similar to those of pesticide toxicity. In this study, sprayers used additional pesticides (e.g., insecticides) for rice, and glyphosate was mostly used for cassava. The type of task and chemicals used are important factors in regard to adverse effects, but it is difficult to identify adverse effects because of mixing. Aspects of participants’ personal hygiene that had health implications included eating in the spraying area, washing hands, showering after work, and cleaning backpack sprayers.

When calculating the internal doses, an individual daily urine output volume of 2 L was assumed, based on the recognized mean daily urine excretion of 1.5–2 L [19]. The internal daily doses of all participants were lower than the RfD, which was assumed to be 1 mg/kg b.w./day based on the U.S. EPA standard [12]. Previously published reports on the internal dose of glyphosate sprayers in the inhalation and dermal exposure groups showed ratios of 0.139–1.165 μg/kg b.w./day and 0.214–4.736 μg/kg b.w./day, respectively [35,36]. One family-farm exposure study reported that the highest estimated internal dose was 0.0006 mg/kg b.w./day [19]. More than half of the participants had no symptoms after application, and showed low internal dose values concordantly. The risk assessments among participants indicated an HQ value of more than 1, which is considered unacceptable and indicates a very high public health risk. Among farm workers, the HQ, which is used to assess risk, was 0.0006 for glyphosate and 0.93 for parathion [37].

This study showed a good correlation between the health risk assessment model and the biomatrix risk assessment model. The health risk assessment matrix methodology has been modified to make it easier for workers to estimate their risk by recognizing the amount of glyphosate, selecting appropriate PPE, and observing adverse effects themselves through the information included in the assessment matrix, as explored in our previous study [15]. Although Thai legislation limits the amount of glyphosate that can be used, there is no quality control regarding the sale, advertisement, or disposal of registered pesticides after they have been registered [38]. Companies expanded pesticide imports to replenish stocks before the ban, and they continue to sell the chemicals despite the prohibition because of quality control issues [39]. In terms of labor rules, the vast majority of Thai agricultural laborers (more than 93%) are undocumented employees who are not covered by Thai labor laws and are not protected by labor, health, and safety laws [39,40]. Farmers’ exposure to pesticides can also be reduced through training programs [40].

It is important to note that due to limitations related to cost and time, the sample size in this study (*n* = 15) was small, and the data analysis was based on only nine cases. Therefore, it is recommended that the sample size be increased in future studies to better explain and estimate the health risk levels of various biomarker concentrations found in chemical sprayers by using biological monitoring. This study found a strong correlation between the risk level determined by the biomatrix of health risk assessment (HRAB), based on the glyphosate biomarker, and the health risk identified by the health risk matrix (HRA) model. This correlation took into account the likelihood of exposure, calculated from the quantity of glyphosate used and the usage of personal protective equipment (PPE). The simplified health risk assessment can be used for easy self-assessment of risk in preventive actions related to health risk awareness among sprayers. In the development and implementation of this modified health risk matrix, regular reviews and updates should be conducted to incorporate new information and technology for the effectiveness of the risk assessment process. Additionally, workers need more knowledge and guidance on how to identify symptoms of adverse effects and determine the types of PPE required for different levels of glyphosate exposure. Training sessions on how to use the modified matrix effectively, coupled with the subsequent development of a sprayer-friendly risk assessment tool, like a mobile application that complements the modified matrix, can assist sprayers in real-time situations and contribute to its successful implementation.

## 5. Conclusions

The concentrations of urinary excreted glyphosate, as internal daily doses among occupational sprayers, were lower than the values indicated by health-based guidance (RfD). The HQ was less than 1, suggesting no significant risk among sprayers. Although the health risk assessment matrix based on exposure to glyphosate and PPE usage and the biomatrix model based on urinary glyphosate concentration (C_urine_) showed that the majority of sprayers were at an acceptable risk level, sprayers should be made more aware of the benefits of using proper PPE for protecting themselves. The Thai government could play a key role in establishing policies and regulations as well as encouraging the safe use of pesticides among sprayers. The next study should develop this health risk assessment matrix tool to allow quick estimation of health risk through a self-assessment program for glyphosate sprayers and raise awareness about the importance of wearing suitable PPE for worker safety.

## Figures and Tables

**Table 1:** Health Risk Assessment (HRA) Matrix and HRA Biomatrix (HRAB) Models (*n* = 15).

Score ^a^	1	2	3	4
Glyphosate exposure (mL)	<100	100–499	500–1000	>1000
Score ^b^	**0**	**1**	**2**	**3**
PPE usage	At least four types of PPE comprised rubber gloves N95/carbon mask, and two other types	At least four types of PPE comprised any type of glove and three other types	At least three types of PPE comprised any type of glove and two other types	At least two types of PPE comprised any type of glove, mask, or other type of PPE
Likelihood ^1^	Low (score = 1–2) (level 1)	Slight (score = 3–4) (level 2)	Moderate (score = 5–6) (level 3)	High (score = 7) (level 4)
Number (%)	-	13 (86.67)	2 (13.33)	-
C_urine_ (μg/L)	≤0.1	>0.1–5.83	>5.84–12.11	>12.11
Likelihood ^2^	Low (level 1)	Slight (level 2)	Moderate (level 3)	High (level 4)
Number (%)	6 (40.00)	2 (13.33)	3 (20.00)	4 (26.67)

Score ^a^: score of glyphosate exposure (mL); Score ^b^: score of PPE usage. Likelihood 1: four levels of scoring according to exposure to an amount of pure glyphosate multiplied by spraying frequency per year plus personal protective equipment (PPE) usage. Likelihood 2: four levels of scoring according to the urinary concentration of glyphosate, C_urine_ (μg/L).

**Table 2:** Descriptive and summary statistics of demographic and task characteristics.

Characteristic of Sprayers	Mean (SD); Min–Max
Age (years)	53.4 (8.4); 36–68
BMI	22.69 (3.40); 19.0–29.7
Experience (years)	16.47 (7.52); 6–30
Frequency of spraying (times/year)	17.07 (16.90); 1–60
Glyphosate volume mixed and sprayed (Milliliters; mL per year)	481.33 (289.87); 100–1000
Duration of spraying (minutes/activity)	180 (143.43); 60–480

**Table 3:** Relationship between behavior when using personal protective equipment (PPE) and symptoms associated with pesticide poisoning.

Parameter	Symptoms Associated with Pesticide Poisoning					
	Symptoms (*n* = 6) N (%)			No-Symptoms (*n* = 9) N (%)		
Gloves	Rubber	Cotton	None	Rubber	Cotton	None
	5 (83.33)	1 (16.67)	-	6 (66.67)	1 (11.11)	2 (22.22)
Mask	Surgical	Cotton	Carbon	Surgical	Cotton	Carbon
	-	5 (83.33)	1 (16.67)	2 (22.22)	7 (77.78)	-
Glasses	Worn	Not worn		Worn	Not worn	
	3 (50.00)	3 (50.00)	-	-	9 (100.00)	-
Hat	Worn	Not worn		Worn	Not worn	
	1 (16.67)	5 (83.33)	-	4 (44.44)	5 (55.56)	-
Boots	Worn	Not worn		Worn	Not worn	
	6 (100.00)	-	-	7 (77.78)	2 (22.22)	-
Long-sleeved shirt and long pants	Worn	Not worn		Worn	Not worn	
	6 (100.00)	-		8 (88.89)	1 (11.11)	

**Table 4:** Personal hygiene of participants with symptoms associated with pesticide poisoning.

Parameter	Symptoms Associated with Pesticide Poisoning			
	Symptoms (*n* = 6) N (%)		No-Symptoms (*n* = 9) N (%)	
	Yes/Sometimes	No	Yes/Sometimes	No
Eating in the spraying area	-	6 (100.00)	3 (33.33)	6 (66.67)
Washing hands 1 h after work	5 (83.33)	1 (16.67)	9 (100.00)	-
Showering 1 h after work	6 (100.00)	-	8 (88.89)	1 (11.11)
Clothing contamination	5 (83.33)	1 (16.67)	4 (44.44)	5 (55.56)
Cleaning backpack sprayers	6 (100.00)	-	8 (88.89)	1 (11.11)

**Table 5:** Health risk calculated by using the likelihood of exposure to glyphosate and PPE usage models and the biomatrix risk assessment model of C_urine_ (*n* = 15).

Severity of Symptoms	Likelihood of Exposure according to the Quantity of Glyphosate and PPE Usage (Health Risk Assessment Matrix: HRA) Model; Number (%)				Likelihood of Exposure according to C_urine_ from the Biomatrix Health Risk Assessment (HRAB) Model; Number (%)			
	Low (1)	Slight (2)	Moderate (3)	High (4)	Low (1)	Slight (2)	Moderate (3)	High (4)
Severe (4)	-	-	-	-	-	-	-	-
Moderate (3)	-	-	-	-	-	-	-	-
Mild (2)	-	6 (40.00)		-	-	-	3 (20.00)	3 (20.00)
None (1)	-	7 (46.67)	2 (13.33)	-	6 (40.00)	2 (13.33)	-	1 (6.67)

There was a good correlation between risk levels from the likelihood of exposure to glyphosate and PPE usage model and risk levels from the biomatrix risk model at a *p*-value < 0.001, r = 0.854.

## Data Availability

Data are contained within the article.
